# *MALAT1*–*miR663a* negative feedback loop in colon cancer cell functions through direct miRNA–lncRNA binding

**DOI:** 10.1038/s41419-018-0925-y

**Published:** 2018-08-28

**Authors:** Wei Tian, Yantao Du, Yuwan Ma, Liankun Gu, Jing Zhou, Dajun Deng

**Affiliations:** Key Laboratory of Carcinogenesis and Translational Research (Ministry of Education/Beijing), Division of Etiology, Peking University Cancer Hospital & Institute, Fu-Cheng-Lu #52, Beijing, 100142 Haidian District China

## Abstract

The lncRNA *MALAT1* has multiple biological functions, including influencing RNA processing, miRNA sponging, and cancer development. It is acknowledged that *miR663a* and its targets are inflammation-related genes frequently deregulated in many cancers. The associations between *MALAT1* and *miR663a* and their target genes remain unknown. In this study, it was found that in colon cancer (CC) cells, *MALAT1* and *miR663a* were reciprocally repressed in cDNA array screening and qRT-PCR analysis. However, *MALAT1* was significantly upregulated in CC tissues, and *miR663a* was significantly downregulated relative to the corresponding surgical margin (SM) tissues. An inverse relationship between *MALAT1* and *miR663a* expression was detected among CC tissue samples (*n* = 172, *r* = −0.333, *p* < 0.0001). The RNA-pulldown results showed *MALAT1* lncRNA–*miR663a* binding. The results of luciferase-reporter analysis further revealed that the *MALAT1* 7038–7059 nt fragment was the *miR663a* seed sequence. Both *miR663a* knockdown and *MALAT1* activation alone significantly upregulated the expression levels of *miR663a* targets, including *TGFB1*, *PIK3CD*, *P53*, *P21*, and *JUND*, in the CC cell lines HCT116 and SW480. A positive relationship was also observed between the expression levels of *MALAT1* and these *miR663a* targets in the above 172 CC samples and 160 CC samples in publicly available databases. In addition, reciprocal abolishment of the effects of *miR663a* overexpression and *MALAT1* activation on the proliferation, migration, and invasion of cancer cells was also observed, while *miR663a* upregulation and *MALAT1* activation alone inhibited and promoted the behaviors of these CC cell lines, respectively. All these suggested that, as a competing endogenous lncRNA, *MALAT1* maybe a dominant protector for the degradation of *miR663a* targets. *miR663a* and *MALAT1* may consist of a negative feedback loop to determine their roles in CC development.

## Background

In the genomes of mammals, less than 2% of the human genome comprises protein-coding sequences, and 98% comprises non-protein coding regions that may play roles in physiological and pathological processes^1^. Long non-coding RNAs (lncRNAs) are transcribed from non-protein coding regions that are longer than 200 nucleotides in length. With the discovery of thousands of lncRNAs, a few have been shown to participate in different biological processes through lncRNA–DNA, lncRNA–RNA, and lncRNA–protein interactions^2^. The *metastasis-associated lung adenocarcinoma transcript 1* gene (*MALAT1, NEAT2*) is the first discovered human tumor-related lncRNA that promotes the progression and metastasis of cancers^3^. *MALAT1* is ubiquitously expressed in normal tissues and is frequently upregulated in human cancers^4–8^. It may function as a component of RNA-splicing nuclear speckles or as a competing endogenous RNA (ceRNA)^9^.

*miR663a* is an inflammation-related miRNA that is frequently deregulated in human cancers. Although the expression level of *miR663a* was significantly upregulated in prostate and nasopharynx cancers, it was markedly downregulated in brain and pancreatic cancers^10–14^. It was reported that *miR663a* inhibited the growth of colon cancer (CC) cells^15^. Although several protein-coding genes have been reported to be *miR663a* targets, upstream networks regulating *miR663a* functions and the mechanisms underlying the effects of *miR663a* on CC development and progression are unclear.

In the present study, we found for the first time that *MALAT1* and *miR663a* directly interacted with, and reciprocally repressed each other. Most importantly, we found that *MALAT1* is a dominant inhibitor of *miR663a* function through preventing the degradation of most *miR663a* targets that are involved in CC development.

## Results

### *MALAT1* expression is mostly decreased by *miR663a* in CC cells

**Fig. 1:**
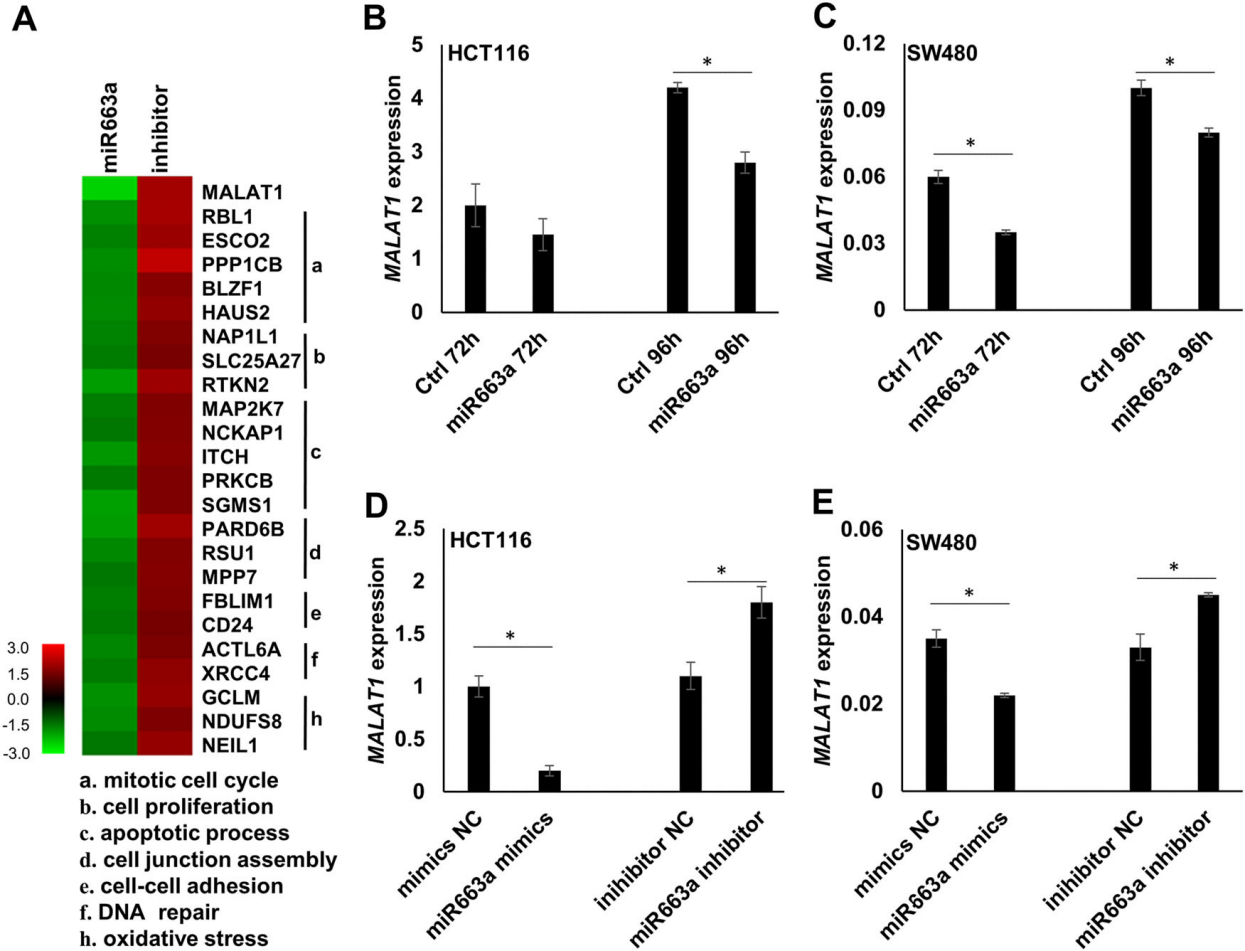
*miR663a* regulates the *MALAT1* expression levels in HCT116 cells and SW480 cells. **a** Heatmap of the expression levels of *MALAT1* and other genes regulated by *miR663a* in cDNA assay analysis and GO_names from gene ontology analysis of the protein-coding genes regulated by *miR663a* [Criteria for selection of these genes: absolute value of fold change >1.5 and *p*-value in gene ontology analysis <0.05]. **b**, **c** qRT-PCR results to detect the *MALAT1* expression changes following transient transfection with the *miR663a* expression vector or control vector in HCT116 and SW480 cells. **d**, **e** qRT-PCR results to detect the *MALAT1* expression changes following transient transfection with the *miR663a* mimics and inhibitor in HCT116 and SW480 cells. *p < 0.05 using Student’s *t*-test

To screen *miR663a* target genes, we performed cDNA microarray analyses using CC HCT116 cells at 72 h after transfection with the *miR663a* expression vector and its inhibitor (antisense), respectively. Using 1.5-fold change as the cutoff value, the mRNA levels of 75 different transcripts from 71 genes were decreased by *miR663a* overexpression and increased by the *miR663a* inhibitor-knockdown. Gene ontology analysis showed that these genes were related to the mitotic cell cycle, cell proliferation, apoptotic process, cell junction assembly, cell–cell adhesion, DNA repair, and oxidative stress (Fig. 1a). Notably, *MALAT1* was mostly downregulated (−3.4-fold) and mostly upregulated (+2.6-fold) among the top 71 genes (Supplemental data file 1).

The effects of *miR663a* expression changes on the *MALAT1* expression level was further confirmed by qRT-PCR analysis. The *MALAT1* level was significantly decreased after transfection with the *miR663a* expression vector in HCT116 and SW480 cells (Fig. 1b, c). Similarly, *MALAT1* downregulation and upregulation were also observed in these cells transiently transfected with the *miR663a* mimics and inhibitor, respectively (Fig. 1d, e). These results suggest that *MALAT1* may be a *miR663a* target.

### *MALAT1* decreases *miR663a* expression in CC cells

**Fig. 2:**
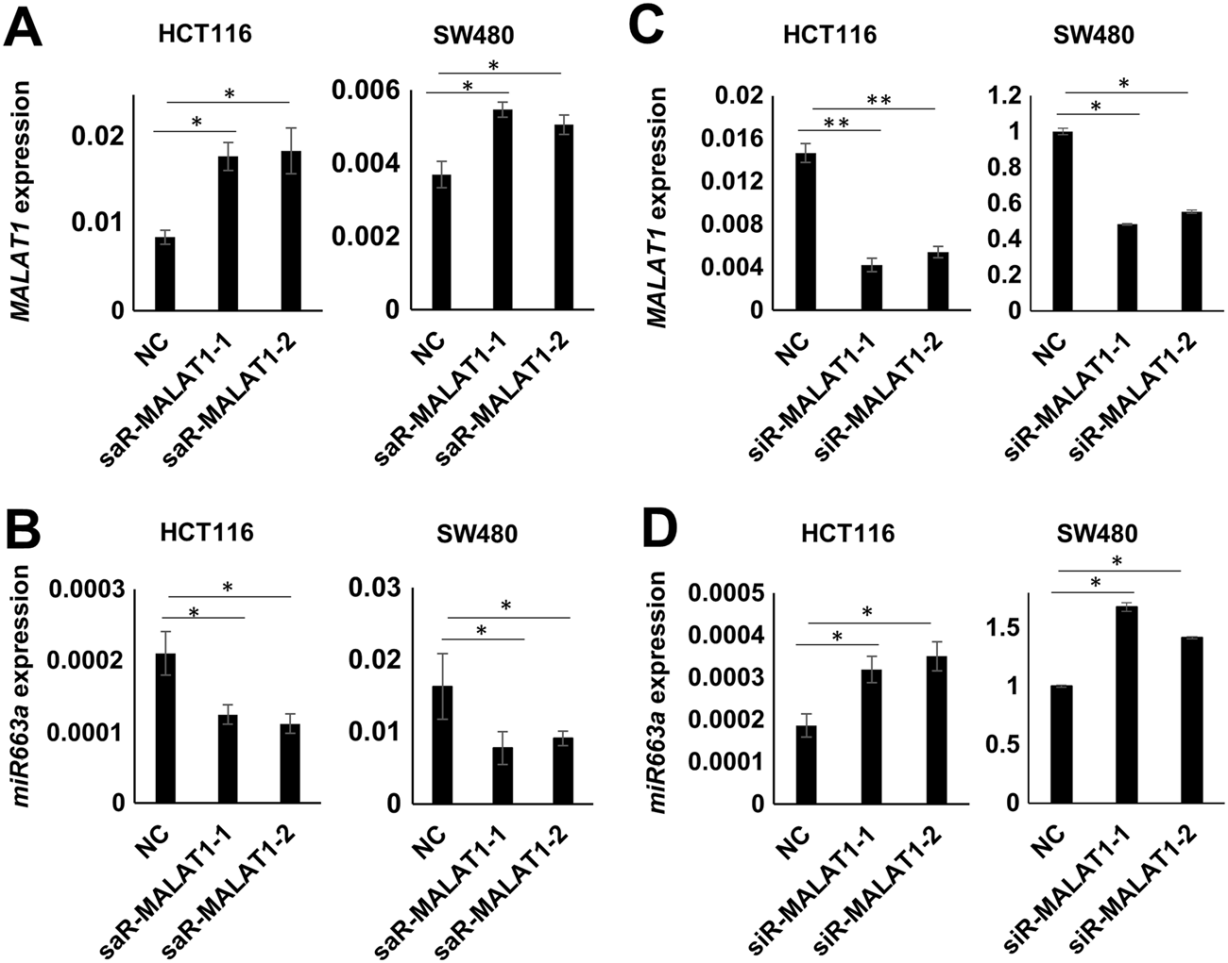
Effects of *MALAT1* expression changes on the *miR663a* expression levels in HCT116 and SW480 cells. **a**, **b** Effects of *MALAT1* upregulation by saR-*MALAT1*-1/-2 on *miR663a* expression. **c**, **d** Effects of *MALAT1* downregulation by siR-*MALAT1*-1/-2 on *miR663a* expression.*p < 0.05 and **p  < 0.01 using Student’s *t*-test

The effects of *MALAT1* expression changes on *miR663a* expression were further studied. Because the full-length sequence of *MALAT1* is >8000 bp, it is difficult to construct a full-length *MALAT1* expression vector. Hence, two small activating RNAs (saR-*MALAT1*-1/-2) complementarily paired to the *MALAT1* promoter sequence were synthesized and used to trigger endogenous *MALAT1* expression in HCT116 and SW480 cells as previously reported^16,17^. As expected, the endogenous *MALAT1* expression level was increased at 72 h following small activation RNA (saRNA) transfection (Fig. 2a). Interestingly, the *miR663a* expression level was consistently and significantly decreased in these cell lines following saR-*MALAT1*-1/-2 transfection (Fig. 2b). In contrast, knockdown of *MALAT1* expression by siR-*MALAT1*-1/-2 remarkably induced *miR663a* upregulation (Fig. 2c, d). These results support that *MALAT1* and *miR663a* maybe reciprocally repressed.

### Inverse relationship between *MALAT1* lncRNA and *miR663a* expression in CC tissues

**Fig. 3:**
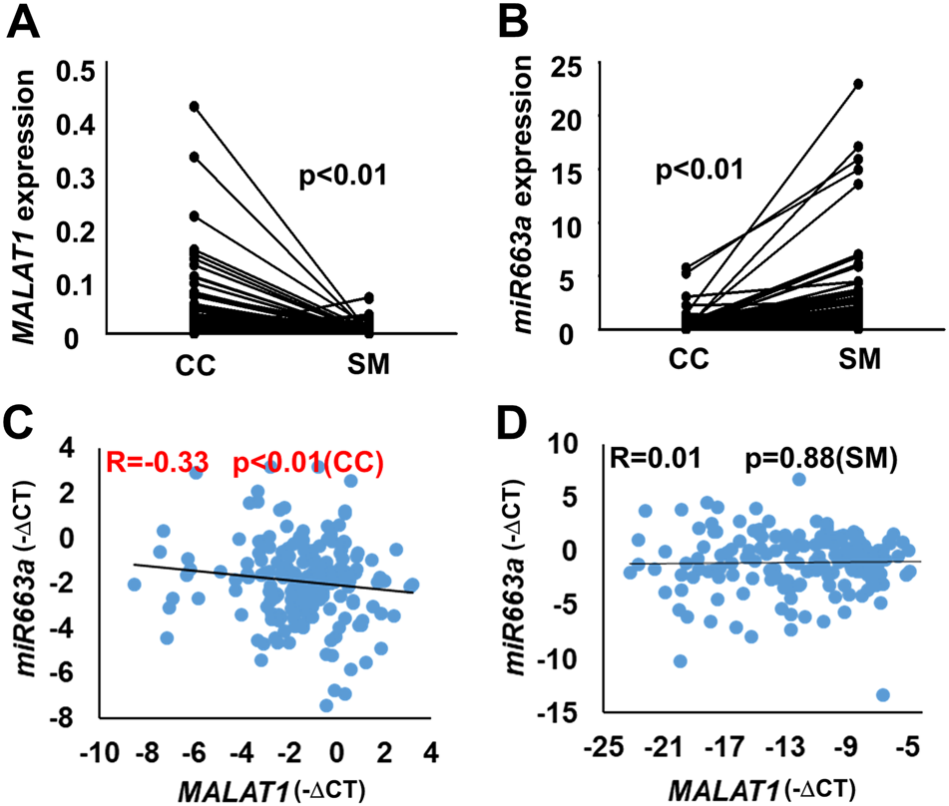
*MALAT1* is negatively related to *miR663a* in colon cancer tissues from 172 patients. **a**, **b** Inverse changes in *MALAT1* and *miR663a* expression between colon cancer (CC) and paired non-cancerous surgical margin (SM) samples. *p*-Values were calculated using the Wilcoxon signed-rank test. **c** Inverse relationship between *MALAT1* and *miR663a* expression in colon cancer tissues, as detected by qRT-PCR. The correlation coefficient and *p*-value were calculated using Spearman’s rank correlation analysis. **d** No significant relationship was found between *MALAT1* and *miR663a* expression in the non-cancerous surgical margin (SM) samples, as detected by qRT-PCR

To confirm the relationship between *MALAT1* and *miR663a* expression in tissues, the expression levels of *MALAT1* and *miR663a* in CC and surgical margin (SM) tissue samples from 172 patients were analyzed by qRT-PCR. It was found that, relative to SM samples, *MALAT1* expression was significantly upregulated (*p* < 0.001; Fig. 3a) while *miR663a* expression was significantly downregulated in CC samples (*p* < 0.001; Fig. 3b). Notably, a significantly inverse relationship between these two genes was also observed in CC samples (*r* = −0.333; *p* < 0.0001; *n* = 172) (Fig. 3c). However, such a relationship was not detected in an equal number of SM samples (Fig. 3d). Together, the above results strongly support that the *miR663a* and *MALAT1* expression levels were reciprocally repressed and involved in CC development.

### *MALAT1* directly interacts with *miR663a*

**Fig. 4:**
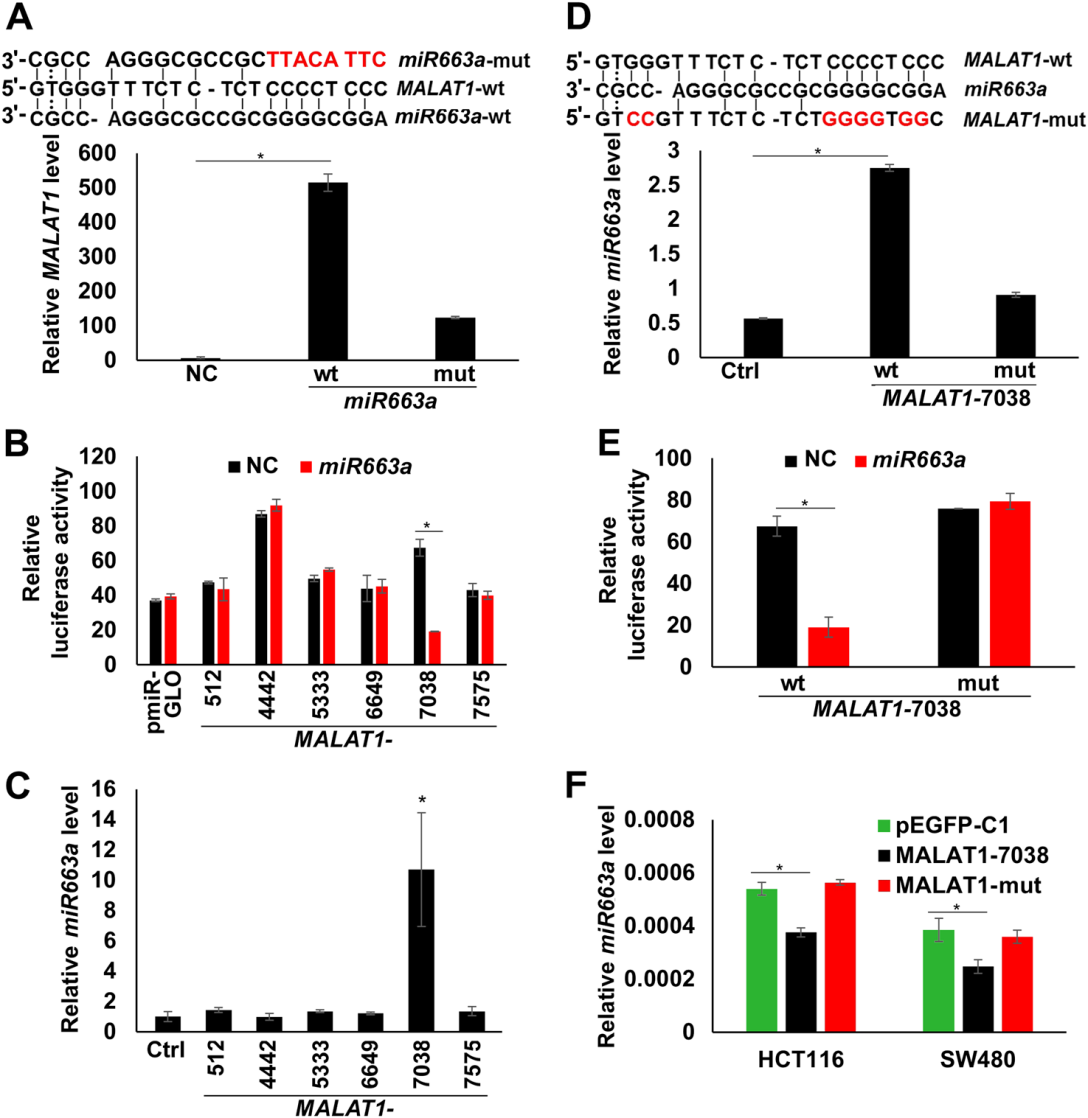
*miR663a* directly binds and inhibits *MALAT1* RNA. **a** Binding of wildtype *miR663a* and its corresponding mutant to *MALAT1* in the RNA pull-down assay using *GAPDH* as an internal reference. Sequences of the *miR663a* candidate target (7038–7059 nt) within *MALAT1* and *miR663a* mutant are listed. **b** Influence of *miR663a* on the luciferase activity of various pmiR-GLO-MALAT1 fragments. **c**, **d** Binding of various wild-type *MALAT1* fragments and the corresponding mutant for *MALAT1-7038* to *miR663a* in the RNA-pulldown assay using *U6* as an internal reference. Sequences of the *miR663a* seed site within *MALAT1* and its mutant are listed. **e** Mutation of the 7038–7059 nt seed site within *MALAT1* abolished the influence of *miR663a* on the pmiRGLO-MALAT1-7038 luciferase activity. **f** The expression status of endogenous *miR663a* in HCT116 and SW480 cells transfected with MALAT1-7038 and its mutant expression vector for 72 h. *p < 0.05 using Student’s *t*-test

*MALAT1* is a well-known *miRNA* sponge. To investigate whether *MALAT1* is also a *miR663a* sponge, the RNA-pulldown assay was carried out. It was found that biotin-labeled *miR663a*-wt pulled down *MALAT1* RNA in HCT116 cells, but biotin-labeled *miR663a*-mut containing a mutant 5′-end did not (Fig. 4a). This indicates that *miR663a* may directly bind to *MALAT1* RNA through its 5′ sequence.

In bioinformatics analysis (RNA22v2), six *miR663a* candidate seed sequences were found within *MALAT1* RNA (Supplemental data file-2), with a high absolute value of folding energy and low *p-*value^18^. To characterize *miR663a*-binding sequence(s) within *MALAT1* RNA, six DNA fragments (approximately 500 bp, each containing one seed sequence) were synthesized, inserted into the pmiR-GLO vector, and used in dual-luciferase reporter analysis. The results showed that *miR663a* only decreased the reporter activity of the *MALAT1*-7038 fragment (containing the 7038–7059 nt seed sequence; Fig. 4b). Similarly, *miR663a* was only pulled down by the biotin-labeled *MALAT1*-7038-wt fragment, but not by other five fragments (Fig. 4c), nor by the biotin-labeled *MALAT1*-7038-mut fragment containing a mutant seed sequence in RNA-pulldown assay in HCT116 cells (Fig. 4d). Mutation of the seed sequence completely abolished the influence of *miR663a* on the *MALAT1* reporter activity (Fig. 4e). In addition, relative to the pEGFP-C1 control vector, the endogenous *miR663a* expression levels were significantly repressed in both HCT116 and SW480 cells transfected with the *MALAT1*-7038-wt vector but not with the *MALAT1*-7038-mut vector (Fig. 4f). The above results demonstrated that *miR663a* could directly bind to the *MALAT1*-7038 seeding site.

### As a master ceRNA, *MALAT1* dominantly prevents the degradation of most *miR663a* targets

**Fig. 5:**
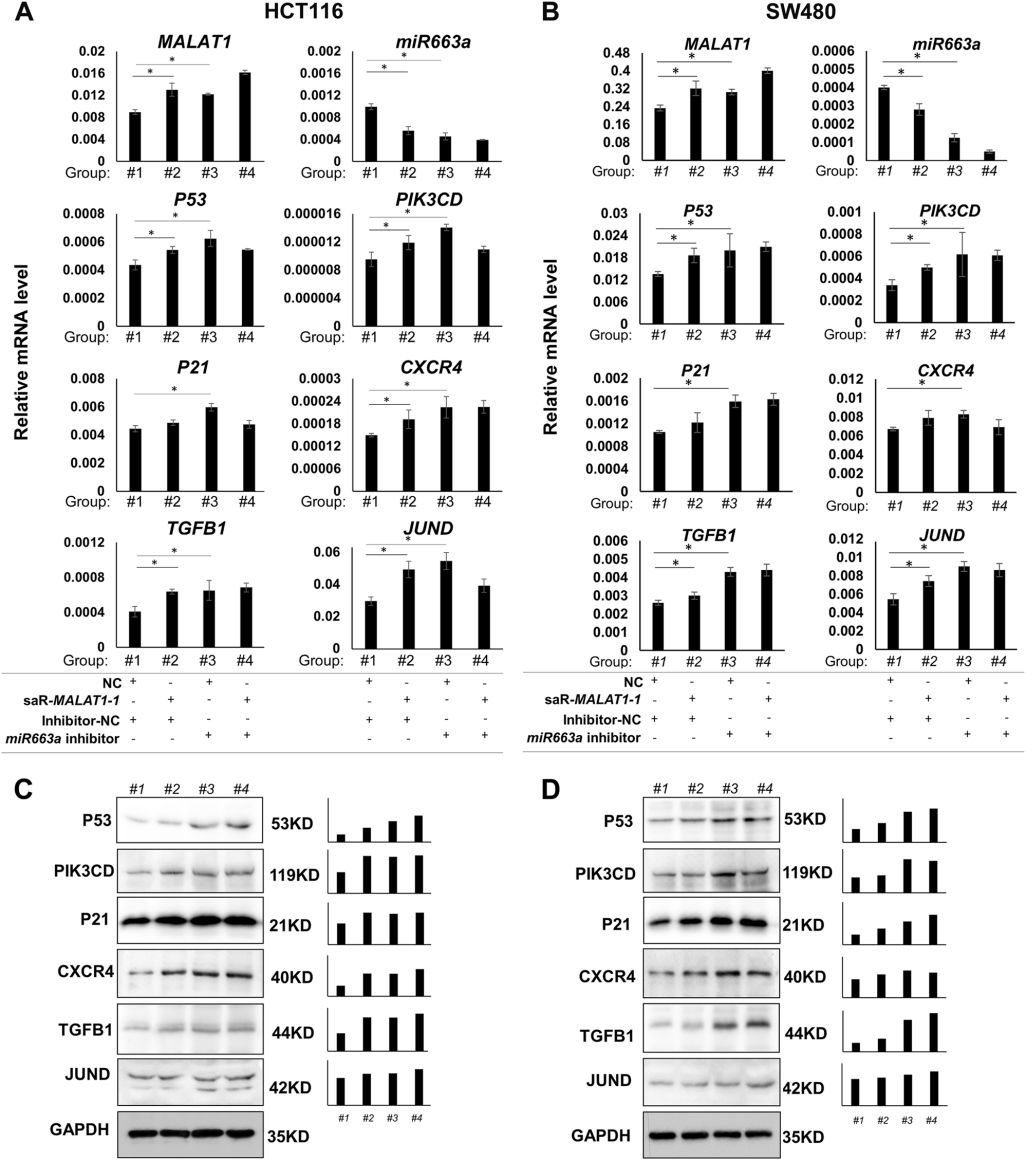
Expression changes of *miR663a* target genes in colon cancer cells transiently transfected with saR-MALAT1-1, *miR663a* inhibitor, and negative control (NC). **a** RT-PCR for HCT116 cells. **b** RT-PCR for SW480 cells. **c**, **d** Western blotting for HCT116 cells and SW480 cells. The density ratio of target proteins to GAPDH reference for each group was also listed in right charts. *p < 0.05 using Student’s *t*-test

To evaluate the significance of *miR663a* downregulation by *MALAT1*, the expression changes of a set of *miR663a* target genes, including *P53*^19^, *PIK3CD*^12^, *P21*^11^, *CXCR4*^13^, *TGFB1*^20^, and *JUND*^21^, were further studied in HCT116 cells. As expected, *miR663a* downregulation by the inhibitor significantly increased the expression levels of these genes (Fig. 5a, #1 vs #3). Similarly, *MALAT1* activation by saR-MALAT1-1 significantly increased the expression levels of most of these genes, except *P21* (Fig. 5a, #1 vs #2). However, when *miR663a* was knocked down by the inhibitor, *MALAT1* activation could no longer increase the expression levels of these genes (Fig. 5a, #3 vs #4). Similar results were also observed in SW480 cells (Fig. 5b). The results of Western blotting analysis confirmed the expression changes of these target genes in HCT116 and SW480 cells. Both *MALAT1* activation and *miR663a* downregulation increased the protein levels of most *miR663a* targets, including P53, PIK3CD, P21, CXCR4, and TGFB1 (Fig. 5c, d, #1 vs #2 and #3). These results suggest that *MALAT1* may dominantly prevent the degradation of *miR663a* target genes in a *miR663a*-dependent pattern.

**Fig. 6:**
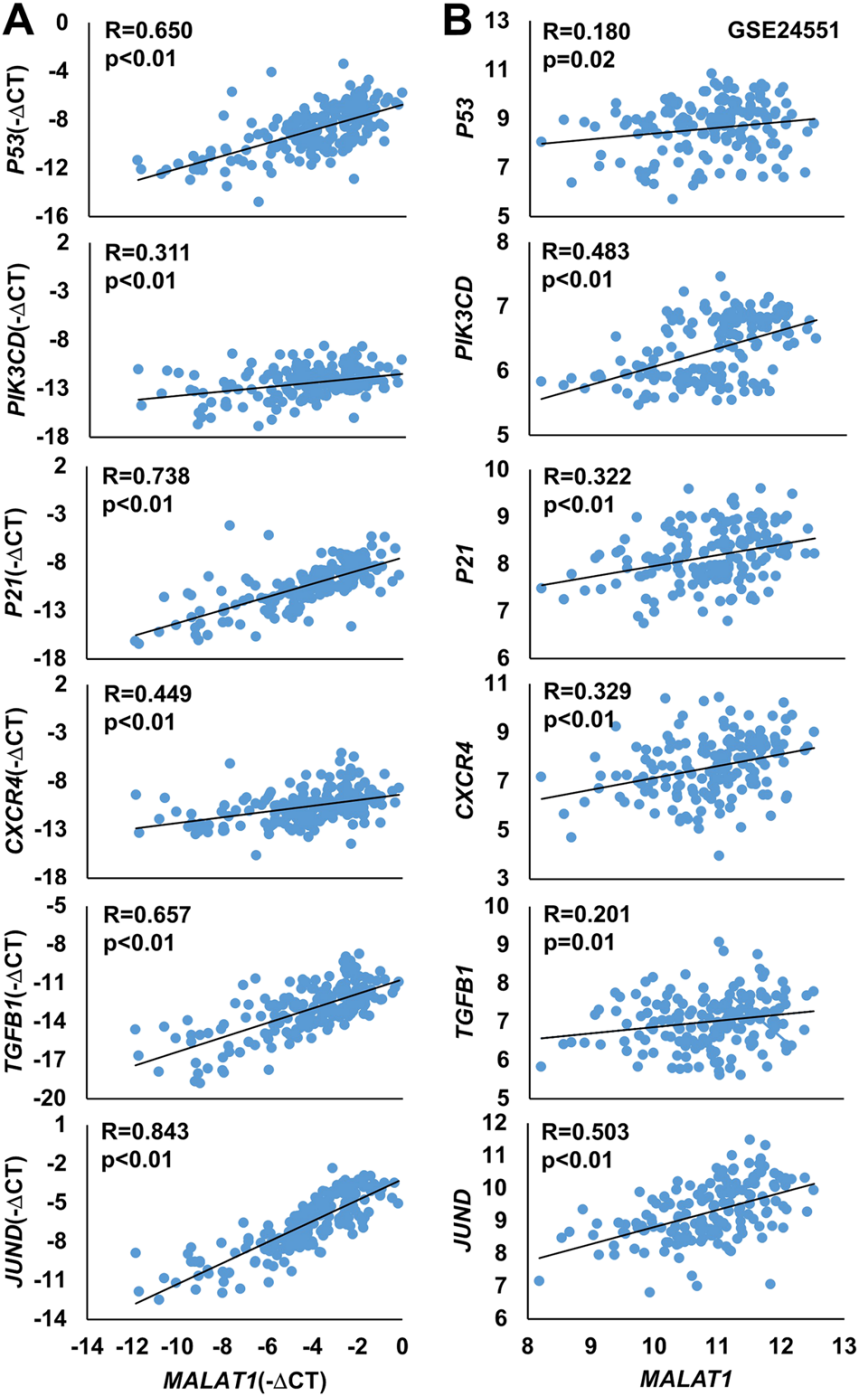
The *MALAT1* lncRNA levels are positively correlated with the mRNA levels of *miR663a* target genes in colon cancer tissues. **a** Colon cancer patients (*n* = 172) listed in Table S1. **b** Colon cancer patients (*n* = 160; from the publicly available database GSE24551). The correlation coefficient and *p*-value were calculated using Spearman’s rank correlation analysis

To confirm the positive relationships between the expression of *MALAT1* and *miR663a* targets, we analyzed the correlation between the *MALAT1* and *miR663a* target expression levels among 172 CC patients described above. As expected, the *MALAT1* expression level was significantly and positively correlated with expression levels of all these genes, including *P53*, *PIK3CD*, *P21*, *CXCR4, TGFB1*, and *JUND* (Fig. 6a). To validate these positive relationships, the publicly available cDNA array datasets for primary colorectal cancers (*n* = 160; GEO GSE24551) were re-analyzed. Once again, the expression levels of almost all *miR663a* targets were all significantly and positively correlated with the *MALAT1* RNA levels in these tissues (Fig. 6b). Collectively, the above results indicate that *MALAT1* may be a master ceRNA that could greatly control the expression levels of these *miR663a* target genes in CC tissues.

### Reciprocal abolishment of the effects of *miR663a* and *MALAT1* on the behavior of CC cells

**Fig. 7:**
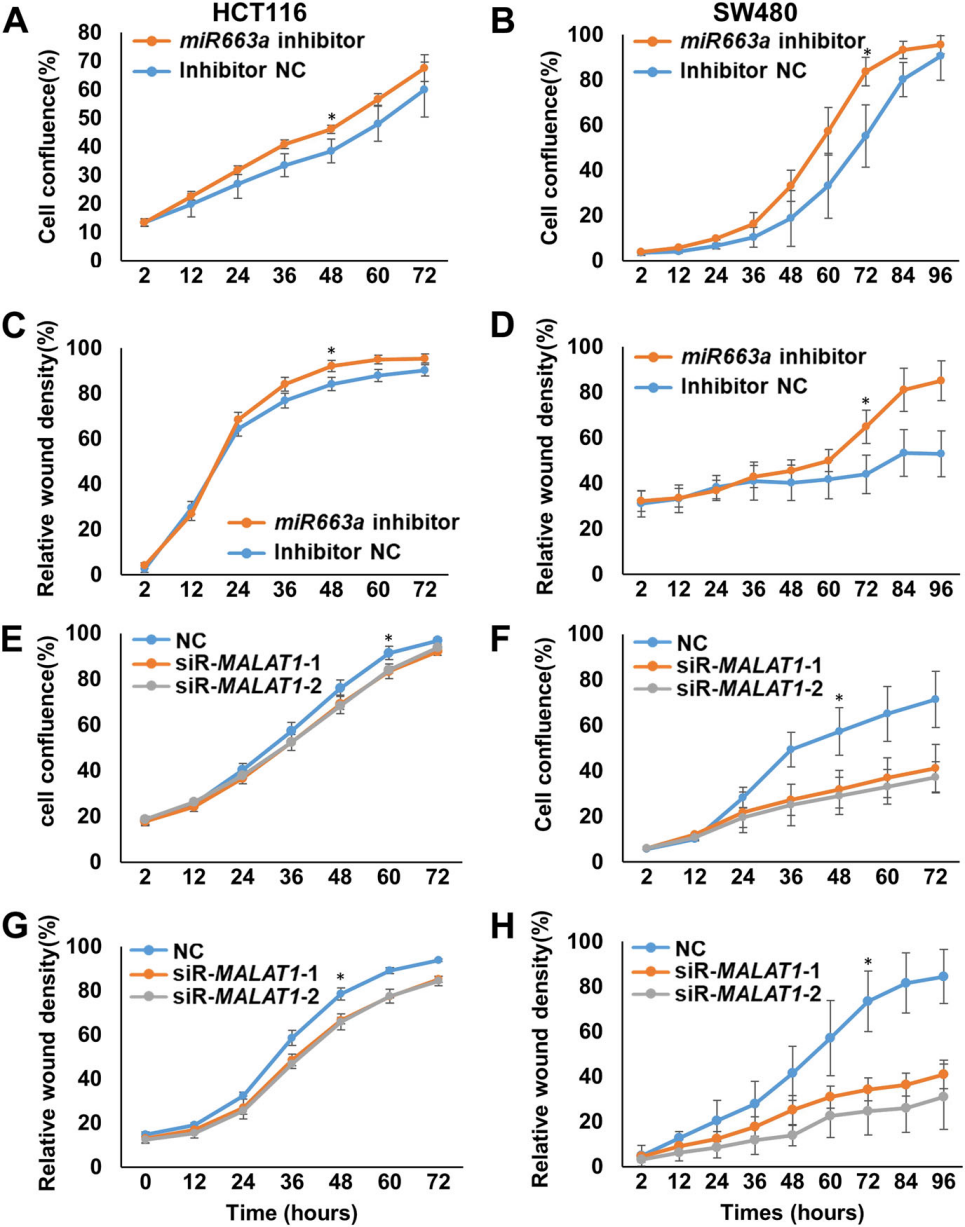
Effects of *MALAT1* and *miR663a* downregulation on the proliferation and migration of colon cancer cells. **a**–**d** Proliferation curve and relative wound density curve for HCT116 and SW480 cells with *miR663a* downregulation by its inhibitor. **e**–**h** Proliferation curve and relative wound density curve for HCT116 and SW480 cells with *MALAT1* downregulation by siR-MALAT1-1/2. *p < 0.05 using Student’s *t*-test

**Fig. 8:**
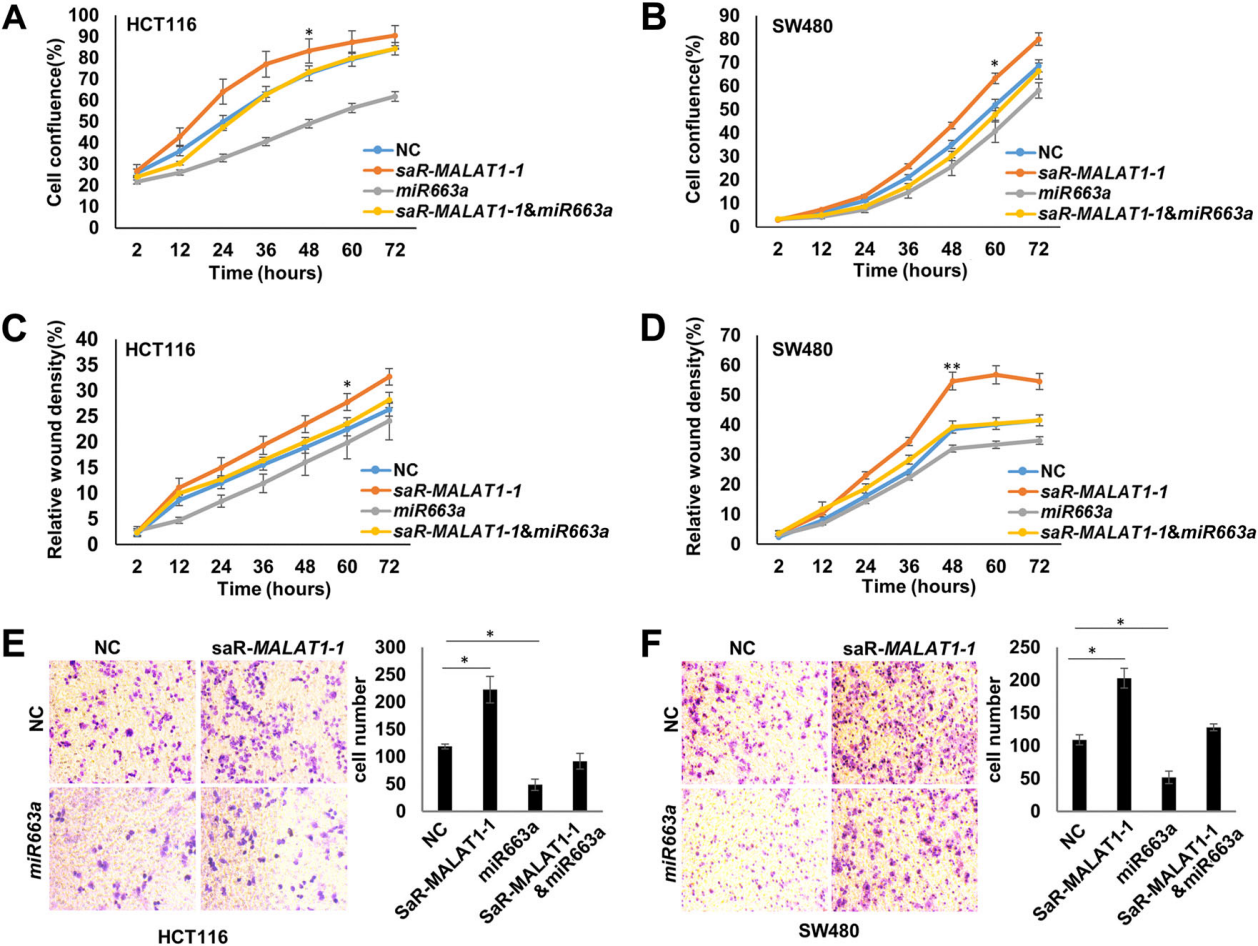
Effects of *MALAT1* activation and *miR663a* overexpression on proliferation, migration, and invasion of colon cancer cells. **a**, **b** Proliferation curve of HCT116 and SW480 cells in IncuCyte long-term dynamic analysis. **c**, **d** Relative wound density curve of HCT116 and SW480 cells in the IncuCyte analysis. **e**, **f** Invasion of HCT116 and SW480 cells in typical Transwell analyses

*MALAT1* is the first characterized oncogenic lncRNA that promotes the progression of many cancers. To evaluate the importance of *miR663a* in the oncogenic effects of *MALAT1* on cancer cells, we performed IncuCyte long-term dynamic proliferation/migration and Transwell invasion analysis. As expected, the downregulation of endogenous *miR663a* by its inhibitor promoted the proliferation and migration of HCT116 and SW480 cells (Fig. 7a–d), while the downregulation of endogenous *MALAT1* by siR-MALAT1-1/2 inhibited the proliferation and migration of these cells (Fig. 7e–h). By contrast, *MALAT1* upregulation by *saR-MALAT1* significantly promoted the proliferation, migration, and invasion of HCT116 and SW480 cells, while *miR663a* overexpression decreased the proliferation, migration, and invasion of these cells (Fig. 8). Interestingly, *MALAT1* upregulation combined with *miR663a* overexpression did not affect the proliferation, migration, and invasion of these cells, indicating reciprocal abolishment of their biological effects on these cells.

## Discussion

*MALAT1*, as a component of nuclear paraspeckles, is a well-studied lncRNA involved in pre-mRNA splicing^22^. It also functions as a miRNA sponge to competitively upregulate miRNA targets^23–26^. Although *MALAT1* is the first characterized oncogenic lncRNA, the mechanisms underlying its effects on cancer development remain unclear^9^. In the present study, we found, for the first time, that *MALAT1* and *miR663a* reciprocally repressed each other through sequence-dependent binding. Moreover, *MALAT1* could competitively prevent the degradation of a set of *miR663a* targets in vitro and in vivo, suggesting it may be a dominant regulator for *miR663a* functions in CC cells. *MALAT1* and *miR663a* may consist of a negative feedback loop and are involved in CC cancer development.

It was reported that the 6918–8441 nt fragment within the *MALAT1* RNA plays a pivotal role in the biological processes of cell proliferation, migration, and invasion of CC cells^27^. We found that the 7038–7059 nt sequence within the 6918–8441 nt fragment is essential for the *miR663a*–*MALAT1* RNA–RNA binding. Mutation of this *miR663a* binding site within *MALAT1* RNA could completely abolish the *miR663a*–*MALAT1* interaction in both the RNA-pulldown and dual-fluorescence reporter assays. Interaction of *miR663a* with other *MALAT1* fragments was not observed. These results demonstrated that the 7038–7059 nt sequence is the only *miR663a* binding site within *MALAT1* RNA and may be an essential point in the feedback loop.

It is well recognized that miRNA promotes the degradation of mRNA through interacting with mRNA 3UTR. Although it is not necessary to expect a ceRNA to control the stability of its interacting molecules, however, the present study indicates that a ceRNA *MALAT1* could promote the endogenous *miR663a* degradation and vice versa. This suggests important effects of the *miR663a*–*MALAT1* loop on the reciprocal regulation of their biological functions.

*MALAT1* may be sheared by endogenous RNase P and RNase Z into a longer segment [1–7072 nt] and a shorter tRNA-like RNA (~60 nt) from the 3′ terminus that would be transported into the cytoplasm^28^. The *miR663a*-binding 7038–7059 nt sequence is located within the longer *MALAT1* segment. Although the longer *MALAT1* segment stays in the nucleus, many reports have shown that *MALAT1* could function as a sponge for many miRNAs^29–31^. In proliferating cells from prophase to anaphase in the cell cycle, disruption of the nuclear membrane leads to merging of the cytoplasm with the nucleus. If nuclear lncRNAs indeed interact with cytoplasmic miRNAs in cells at these mitotic stages, these interactions should be cell proliferation-dependent. This might account for the significantly inverse *miR663a*–*MALAT1* relationship observed in CC tissues containing a high proportion of proliferating cancer cells but not in an equal number of SM samples containing a few proliferating tissue stem cells. It is an important issue to address the possible roles of the disruption of the nuclear membrane in actively proliferating cells in occurrence of interactions between nuclear components and cytoplasm components.

Most importantly, our results revealed that *miR663a* overexpression accompanied with *MALAT1* upregulation did not affect the proliferation, migration, and invasion of CC cells, whereas *miR663a* downregulation alone or *MALAT1* upregulation alone promoted the proliferation, migration, and invasion of CC cells. The *miR663a* gene is a primate-specific gene that is absent in the mouse and rat genomes. To study whether *MALAT1* may affect the proliferation, migration, and invasion of CC cells in a *miR663a-*dependent manner, other animal models should be used.

Chronic inflammation may play a causal role in CC development. It was reported that *MALAT1* expression is increased^32–34^ and *miR663a* expression is decreased in inflammatory tissues^35,36^. We found obvious *MALAT1* overexpression and *miR663a* downregulation in CC tissues. The long-term administration of aspirin, as an antioxidant, decreases CC risk in the population. Antioxidant resveratrol treatment not only could prevent inflammation but could also restore the expression status of *MALAT1* and *miR663a* to normal levels^20,21,37^. It is unknown whether aspirin treatment has similar effects. The effects of re-balancing *MALAT1* and *miR663a* expression by antioxidants on CC development should be studied in the future.

We found that the expression levels of a set of *miR663a* targets were positively and significantly associated with *MALAT1* expression in both 172 CC samples analyzed in the present study and 160 CC samples from public databases and that both *miR663a* knockdown and *MALAT1* activation increased the expression levels of these target genes. These phenomena indicated that *MALAT1* may be a dominant regulator for *miR663a* targets.

In conclusion, we found that *MALAT1* was significantly upregulated and *miR663a* was significantly downregulated in CC tissues. *MALAT1* and *miR663a* could reciprocally repress each other through direct RNA–RNA binding. *MALAT1* might be a dominant regulator for *miR663a* targets through competitive interactions with *miR663a*. *MALAT1* and *miR663a* could reciprocally abolish their biological functions in CC development, which might be a useful intervention target for CC prevention.

## Materials and methods

### Synthesis of RNA nucleotides and plasmids

For *MALAT1* upregulation, saRNA sequences were designed to target the *MALAT1* promoter, including saR-*MALAT1*-1 (574–592 nt; forward: 5′-cgaga auucu agacu aguatt-3′; reverse: 5′-uacua gucua gaauu cucgtt-3′) and saR-*MALAT1*-2 (330–348 nt; forward: 5′-gcaga guagc gaccg agaatt-3′; reverse: 5′-uucuc ggucg cuacu cugctt-3′)^16,17^. For *MALAT1* knockdown, siRNA sequences were siR-*MALAT1*-1 (5098–5116 nt; forward: 5′-gcaaa ugaaa gcuac caau-3′; reverse: 5′-auugg uagcu uucau uugctt-3′) and siR-*MALAT1*-2 (6392–6410 nt; forward: 5′-gcaga ggcau uucau ccuu-3′; reverse: 5′-aagga ugaaa ugccu cugctt-3′).

The wildtype *MALAT1* fragments (approximately 500 bp; Supplemental data file-2) and its *miR663a-*seeding-site mutant were synthesized and inserted into the pmiR-GLO vector. To construct the *MALAT1*-7038 fragment and its mutant expression vectors, the PCR products were amplified from pmiR-GLO-MALAT1 and pmiR-GLO-7038-mutant vectors using a primer set (forward: 5′-ggaaa tttct gcagt tttaa-3′; reverse: 5′-ttcac ctgtt ttcct cattt-3′) and were inserted into the pEGFP-C1 vector.

The sequences of the *miR663a* mimics were as follows: forward: 5′-aggcg gggcg ccgcg ggaccgc-3′; reverse: 5′-ggucc cgcgg cgccc cgccuuu-3′. A scrambled siRNA set (NC, forward 5′-uucuc cgaac guguc acgutt-3′ and reverse 5′-acgug acacg uucgg agaatt-3′) was used as the saRNA/siRNA/mimics negative control. The sequence of the *miR663a* inhibitor (antisense) was 5′-gcggu cccgc ggcgc cccgc cu-3′. A scrambled RNA inhibitor (Inhibitor NC, 5′-cagua cuuuu gugua guacaa-3′) was used as the inhibitor negative control. All these synthesized RNA products were purchased from Genepharma (Shanghai, China). The *miR663a* expression vector pcDNA3.1b-pri-miR663a was constructed using 93 bp PCR products amplified from the genomic DNA of A549 cells using HiFi DNA polymerase (Transgen Biotech, Beijing, China) and the primer set (forward: 5′-ccttc cggcg tccca ggcg-3′; reverse: 5′-catgg ccggg ccacc aggaaa-3′).

### Cells culture and tissue samples

Human CC cell lines HCT116 and SW480 were purchased from the American Type Culture Collection (ATCC, Manassas, USA). These cell lines were cultured in RPMI 1640 medium or DMEM medium containing 10% FBS and 100 U/mL penicillin/streptomycin (Invitrogen, CA, USA) at 37 °C in a humidified incubator with 5% CO_2_. X-tremeGENE siRNA Transfection Reagent or X-tremeGENE HP DNA Transfection Reagent (Roche, Mannheim, Germany) were used in the cell transfection of miRNAs (final concentration, 100 nM) or plasmids (2 μg/well in 6-well plates) following the manufacturer’s instructions. The overexpression or knockdown efficiency was determined after transfection by reverse transcription-PCR (RT-PCR) and Western blotting at the indicated time points.

The CC tissues and paired non-cancerous SM samples were collected from 172 patients (average age, 61.64 years; 101 males and 71 females; 89 CCs at pTNM stage I–II and 83 CCs at the stage III–IV) at the Peking University Cancer Hospital and Institute from 2004 to 2011 (Table S1). The Peking University Cancer Hospital and Institute Review Boards approved this study. All patients provided written informed consent to participate in the study.

### cDNA array and bioinformatics analysis

HCT116 cells were harvested at 72 h after transient transfection of the pcDNA3.1b-pri-miR663a vector, antisense/inhibitor, and corresponding negative controls. Total RNA was extracted with TRIzol reagent (Life Technologies, CA, USA), reverse transcribed to cDNA, and used for Affymetrix U133Plus 2.0 array analysis. The gene expression levels were assessed by the fluorescence signal attached to the probe and then to template strand in PCR. After log transformation, the fold changes were calculated according to the *miR663a* expression vector group vs pcDNA3.1b vector control group, or antisense/inhibition group vs inhibitor NC group. The gene expression levels changed inversely in the above two comparisons were selected as *miR663a* candidate targets (Supplemental data file-1).

### Quantitative RT-PCR

The quality and concentration of RNA samples were monitored using the NanoDrop 2000 system (Thermo Fisher Scientific, Waltham, MA, USA). Qualified RNA samples were used to synthesize cDNA using the TransScript First-Strand cDNA Synthesis SuperMix (TransGen Biotech, Beijing, China). Because of the absence of introns within *MALAT1* RNA, to exclude false *MALAT1*-amplification from DNA templates, the total RNA was pre-digested with DNase before cDNA synthesis. Quantitative RT-PCR (qRT-PCR) was performed using a StepOne Real-time PCR System (Applied Biosystems, Foster City, CA, USA) and SYBR Green PCR master mix reagents (FastStart Universal SYBR Green Master, Roche, Mannheim, Germany). *MALAT1* and the protein-coding gene expression levels were normalized to those of *GAPDH* (for cells) *and Alu* (for tissues). The relative mRNA level was calculated using the classical delta-delta-Ct method. The Bulge-Loop™ miRNA qRT-PCR starter kit (RuiBO, Guangzhou, China) was used to determine the *miR663a* levels, and the *U6* transcription level was used as a reference. The sequences of primers used in these PCR assays are listed in Table S2. Each sample was determined in triplicate.

### RNA-pulldown assay

In total, 3 × 10^6^ HCT116 cells were seeded on the 10-cm plate for 24 h. Next, these cells were transfected with biotin-labeled NC (forward: 5′-uucuc cgaac guguc acgutt-3′; reverse: 5′-acgug acacg uucgg agaatt-3′), biotin-labeled *miR663a*-wt (forward: 5′-aggcg gggcg ccgcg ggacc gc-3′; reverse 5′-gcggu cccgc ggcgc cccgc cu-3′), or biotin-labeled *miR663a*-mut (forward: 5′-cuuac auucg ccgcg ggacc gc-3′; reverse: 5′-gcggu cccgc ggcga augua ag-3′) at a final concentration of 100 nM. The cells were harvested at 48 h post-transfection. Activated Streptavidin-Dyna beads (Dyna beads M-280 Streptavidin, #11205D, Invitrogen) were coated with 10 μL per sample yeast tRNA (10 mg/mL stock; Ambion, Austin, USA) and 10 μL BSA (10 ng/mL stock) and were incubated in the lysis buffer (480 μL) with rotation at 4 °C for 0.5 h. The beads were then washed, and the sample lysates (600 μL) were mixed with pre-coated beads (50 μL per sample) and incubated at 4 °C for 4 h on a rotator. The beads were then pelleted down the next day to remove unbound materials at 4 °C for 2 min, 500*g*, and were washed six times with 500 μL of ice cold lysis buffer. The *MALAT1* levels in the pulldown samples were detected by qRT-PCR and normalized using *GAPDH* as an internal reference.

In the *MALAT1*-pulldown assay, various wild-type *MALAT1* fragments and *MALAT1*-7038 mutant control were amplified from the corresponding pmiR-GLO-*MALAT1* vector using the corresponding primer set (Table S2), digested with restriction enzyme *Sal*I, inserted into the pGEM-T vector, and transcribed by T7 RNA Polymerase using Riboprobe in vitro Transcription Systems (P1460, Promega, Madison, WI, USA). The RNA was labeled with Pierce^TM^ RNA 3′ End Desthiobiotinylation Kit (20163, Thermo Scientific, Rockford, IL, USA). The *MALAT1* binding RNA was captured by magnetic beads and was used to pull down *MALAT1*-interacting RNA as described above. The *miR663a* levels in the pulldown samples were detected by qRT-PCR, and normalized using *U6* as an internal reference.

### Western blotting

The primary monoclonal antibodies used in Western blot analyses were sc-126 for P53, sc-55589 for PIK3CD, sc-53534 for CXCR-4 (Santa Cruz Biotechnology, Inc, Santa Cruz, CA, USA), CST-2947 for P21 (Cell Signaling Technology, Danvers, MA, USA), ab-25121 for TGFB1 (Abcam, Cambridge, UK), QJ221464 for JUND (Thermo Fisher Scientific, Waltham, MA, USA), and 60004-1-Ig for GAPDH (Proteintech, Rosemont, IL, USA). The signals were visualized using the enhanced chemiluminescence kit (Pierce, Rockford, IL, USA).

### Dual-luciferase reporter assay

The pmiR-GLO-MALAT1 and pmiR-GLO-7038-mutant vectors were used to transfect HCT116 cells in 24-well plate. *MiR663a* mimics or NC were transfected on the second day (3 wells/group). At 72 h post-transfection, the activities of both Renilla and firefly luciferases were measured using the Dual-Luciferase Reporter Assay System (Promega, Madison, WI, USA). The results were presented after normalization with the measured values of the firefly luciferase.

### Cell proliferation and migration assays using IncuCyte

HCT116 and SW480 cells were seeded into 96-well plates (3000 cells/well, 5 wells/group), and were cultured for at least 96 h to determine the proliferation curves. The cells were photographed every 12 h in the long-term dynamic observation platform (IncuCyte, Essen, MI, USA). The cell confluence was analyzed using IncuCyte ZOOM software (Essen, Ann Arbor, MI, USA). For continuous observation for cell migration, the cells were seeded into 96-well plates at a density of 10,000 cells/well and were cultured for 24 h. After a wound was scratched, the cells were washed three times with PBS. The cells were regularly cultured and photographed every 12 h for at least 96 h. Both the relative wound density and width were calculated using the same software.

### Transwell assays

Twenty-four-well plates of Transwell permeable, which supports with a 6.5-mm insert and an 8.0-μm polycarbonate membrane (Corning 3422, Kennebunk, ME, USA), were used in the cell invasion assay. The upper chamber was pre-coated with 100 μL of the BD Matrigel mixture (BD Biosciences, San Jose, CA, USA) and 1640 medium (Gibco, Waltham, MA, USA, 1:5, v/v). To the lower chamber was added 800 μL of 1640 with 10% FBS. The HCT116 cells were seeded into the upper chamber (30,000 cells/well, 3 wells/group) with 1640 medium without FBS. After 24 or 36 h of incubation, the 6.5-mm insert was removed from the plate and fixed in 4% paraformaldehyde, followed by staining with crystal violet staining solution. Non-migrated/noninvasive cells on the upper surface of the insert were wiped with a cotton swab. The migrated/invaded cell number was manually counted in four randomly selected fields under a light microscope. All experiments were performed at least three times.

### Statistical analyses

All statistical analyses were performed using SPSS 18.0 software. The Kolmogorov–Smirnov test was used to estimate the normality of distributions. The Mann–Whitney *U*-test was conducted for non-normally distributed data. Student’s *t*-test was conducted for normally distributed data. The relationship between the levels of *MALAT1* and *miR663a* target genes was measured using the nonparametric correlation test and curvilinear regression model. Statistical significance was assigned at *p* < 0.05 (*) or *p* < 0.01 (**). All experiments were performed at least three times with triplicate samples.

## Electronic supplementary material

Supplemental data file-1

Supplemental data file-2

Supplementary materials
