# Supplementary material for: MALAT1–miR663a negative feedback loop in colon cancer cell functions through direct miRNA–lncRNA binding
Source: Cell Death Dis. 2018 Aug 28;9(9):857. doi: 10.1038/s41419-018-0925-y (PMC6113222; doi:10.1038/s41419-018-0925-y)
Supplement: Supplementary file 2 — Supplemental data file-2 [file 41419_2018_925_MOESM2_ESM.doc]

**Supplemental data file2.**

The sequences of *MALAT1* fragments inserted in pmiR-GLO vectors

(The highlighted sequences were the miR663a seeding candidates.)

MALAT1-512 fragment [folding energy, |20.5| kcal/mol (p=7.65E-2)]

AAACTCTGCAGTTTGGTCTTGGGGTTTGGAGGAAAGCTTTTATTTTTCTTCCTGCTCCGGTTCAGAAGGTCTGAAGCTCATACCTAACCAGGCATAACACAGAATCTGCAAAACAAAAACCCCTAAAAAAGCAGACCCAGAGCAGTGTAAACACTTCTGGGTGTGTCCCTGACTGGCTGCCCAAGGTCTCTGTGTCTTCGGAGACAAAGCCATTCGCTTAGTTGGTCTACTTTAAAAGGCCACTTGAACTCGCTTTCCATGGCGATTTGCCTTGTGAGCACTTTCAGGAGAGCCTGGAAGCTGAAAAACGGTAGAAAAATTTCCGTGCGGGCCGTGGGGGGCTGGCGGCAACTGGGGGGCCGCAGATCAGAGTGGGCCACTGGCAGCCAACGGCCCCCGGGGCTCAGGCGGGGAGCAGCTCTGTGGTGTGGGATTGAGGCGTTTTCCAAGAGTGGGTTTTCACGTTTCTAAGATTTCCCAAGCAGACAGCCCGTGCTGCT

MALAT1-4442 fragment [folding energy, |18.6| kcal/mol (p=1.09E-1)]

ATTGCAGATAAACTCATGCCAGAGAACTTAAAGTCTTAGAATGGAAAAAGTAAAGAAATATCAACTTCCAAGTTGGCAAGTAACTCCCAATGATTTAGTTTTTTTCCCCCCAGTTTGAATTGGGAAGCTGGGGGAAGTTAAATATGAGCCACTGGGTGTACCAGTGCATTAATTTGGGCAAGGAAAGTGTCATAATTTGATACTGTATCTGTTTTCCTTCAAAGTATAGAGCTTTTGGGGAAGGAAAGTATTGAACTGGGGGTTGGTCTGGCCTACTGGGCTGACATTAACTACAATTATGGGAAATGCAAAAGTTGTTTGGATATGGTAGTGTGTGGTTCTCTTTTGGAATTTTTTTCAGGTGATTTAATAATAATTTAAAACTACTATAGAAACTGCAGAGCAAAGGAAGTGGCTTAATGATCCTGAAGGGATTTCTTCTGATGGTAGCTT

TTGTATTATCAAGTAAGATTCTATTTTCAGTTGTGTGTAAGCAAGTT

MALAT1-5333 fragment [folding energy, |18.1| kcal/mol (p=3.43E-1)]

GCCAAAAAATTTTAAGCAAATGAAAGCTACCAATTTAAAGTTACGGAATCTACCATTTTAAAGTTAATTGCTTGTCAAGCTATAACCACAAAAATAATGAATTGATGAGAAATACAATGAAGAGGCAATGTCCATCTCAAAATACTGCTTTTACAAAAGCAGAATAAAAGCGAAAAGAAATGAAAATGTTACACTACATTAATCCTGGAATAAAAGAAGCCGAAATAAATGAGAGATGAGTTGGGATCAAGTGGATTGAGGAGGCTGTGCTGTGTGCCAATGTTTCGTTTGCCTCAGACAGGTATCTCTTCGTTATCAGAAGAGTTGCTTCATTTCATCTGGGAGCAGAAAACAGCAGGCAGCTGTTAACAGATAAGTTTAACTTGCATCTGCAGTATTGCATGTTAGGGATAAGTGCTTATTTTTAAGAGCTGTGGAGTTCTTAAATATCAACCATGGCACTTTCTCCTGACCCCTTCCCTAGGGGATTTCAGGATTGA

MALAT1-6649 fragment [folding energy, |19.1| kcal/mol (p=2.69E-2)]

CATTTCATCCTTCATGAAGCCATTCAGGATTTTGAATTGCATATGAGTGCTTGGCTCTTCCTTCTGTTCTAGTGAGTGTATGAGACCTTGCAGTGAGTTTATCAGCATACTCAAAATTTTTTTCCTGGAATTTGGAGGGATGGGAGGAGGGGGTGGGGCTTACTTGTTGTAGCTTTTTTTTTTTTTACAGACTTCACAGAGAATGCAGTTGTCTTGACTTCAGGTCTGTCTGTTCTGTTGGCAAGTAAATGCAGTACTGTTCTGATCCCGCTGCTATTAGAATGCATTGTGAAACGACTGGAGTATGATTAAAAGTTGTGTTCCCCAATGCTTGGAGTAGTGATTGTTGAAGGAAAAAATCCAGCTGAGTGATAAAGGCTGAGTGTTGAGGAAATTTCTGCAGTTTTAAGCAGTCGTATTTGTGATTGAAGCTGAGTACATTTTGCTGGTGTATTTTTAGGTAAAATGCTTTTTGTTCATTTCTGGTGGTGGGAGGGGAC

MALAT1-7038 fragment [folding energy, |16.0| kcal/mol (p=3.36E-3)]

GGAAATTTCTGCAGTTTTAAGCAGTCGTATTTGTGATTGAAGCTGAGTACATTTTGCTGGTGTATTTTTAGGTAAAATGCTTTTTGTTCATTTCTGGTGGTGGGAGGGGACTGAAGCCTTTAGTCTTTTCCAGATGCAACCTTAAAATCAGTGACAAGAAACATTCCAAACAAGCAACAGTCTTCAAGAAATTAAACTGGCAAGTGGAAATGTTTAAACAGTTCAGTGATCTTTAGTGCATTGTTTATGTGTGGGTTTCTCTCTCCCCTCCCTTGGTCTTAATTCTTACATGCAGGAACACTCAGCAGACACACGTATGCGAAGGGCCAGAGAAGCCAGACCCAGTAAGAAAAAATAGCCTATTTACTTTAAATAAACCAAACATTCCATTTTAAATGTGGGGATTGGGAACCACTAGTTCTTTCAGATGGTATTCTTCAGACTATAGAAGGAGCTTCCAGTTGAATTCACCAGTGGACAAAATGAGGAAAACAGGTGAA

MALAT1-7575 fragment [folding energy, |14.2| kcal/mol (p=2.86E-1)]

TTATGGGACAATAGTATTGAATAGATTTCAGCTTTATGCTGGAGTAACTGGCATGTGAGCAAACTGTGTTGGCGTGGGGGTGGAGGGGTGAGGTGGGCGCTAAGCCTTTTTTTAAGATTTTTCAGGTACCCCTCACTAAAGGCACCGAAGGCTTAAAGTAGGACAACCATGGAGCCTTCCTGTGGCAGGAGAGACAACAAAGCGCTATTATCCTAAGGTCAAGAGAAGTGTCAGCCTCACCTGATTTTTATTAGTAATGAGGACTTGCCTCAACTCCCTCTTTCTGGAGTGAAGCATCCGAAGGAATGCTTGAAGTACCCCTGGGCTTCTCTTAACATTTAAGCAAGCTGTTTTTATAGCAGCTCTTAATAATAAAGCCCAAATCTCAAGCGGTGCTTGAAGGGGAGGGAAAGGGGGAAAGCGGGCAACCACTTTTCCCTAGCTTTTCCAGAAGCCTGTTAAAAGCAAGGTCTCCCCACAAGCAACTTCTCTGCCACATC
