# Supplementary material for: MALAT1–miR663a negative feedback loop in colon cancer cell functions through direct miRNA–lncRNA binding
Source: Cell Death Dis. 2018 Aug 28;9(9):857. doi: 10.1038/s41419-018-0925-y (PMC6113222; doi:10.1038/s41419-018-0925-y)
Supplement: Supplementary file 3 — Supplementary materials [file 41419_2018_925_MOESM3_ESM.pdf]

Supplementary materials

Table S1. Clinicopathological characteristics of 172 patients with colon carcinoma

		Case no.	Percentage (%)
Age (years)	≤60	75	43.6
	>60	97	56.4
Sex	Male	101	58.7
	Female	71	41.3
Location	Sigmoid	87	50.6
	Others	85	49.4
Differentiation	Poor	33	19.2
	Mod./Well	139	80.8
Vascular embolus	Negative	140	81.4
	Positive	32	18.6
pTNM stage	I+II	89	51.7
	III+IV	83	48.3
depth of invasion	T_{1-3}	101	58.7
	T_4	71	41.3
lymph metastasis	$Negative_0$	88	51.2
	Positive	84	48.8
distant metastasis	M_0	147	85.5
	M_1	25	14.5

Table S2. Sequences for oligo primers

Gene	Assay	Primer name	Sequence (5'-3')	Product size	PCR Temp (°C)
MALAT1	qRT-PCR	MALAT1-RT-S	GGTAACGATGGTGTCGAGGTC	190 bp	58
		MALAT1-RT-AS	CCAGCATTACAGTTCTTGAACATG		
MALAT1-	qRT-PCR	M7038-wt-S	TCTCTCTCCCCTCCCTTGGT	83 bp	58
7038-wt		M7038-wt-AS	TCTGGCTTCTCTGGCCCTTC		
MALAT1-	qRT-PCR	M7038-mut-S	TCTCTCTGGGGTGGCTTGGT	83 bp	58
7038-mut		M7038-mut-AS	TCTGGCTTCTCTGGCCCTTC		
TGFB1	qRT-PCR	TGFB1-RT-S	AAGGACCTCGGCTGGAAGTG	137 bp	58
		TGFB1-RT-AS	CCCGGGTTATGCTGGTTGTA		
JUNB	qRT-PCR	JUNB-RT-S	ATGGAACAGCCCTTCTACCACG	104 bp	58
		JUNB-RT-AS	AGGCTCGGTTTCAGGAGTTTG		
JUND	qRT-PCR	JUND-RT-S	GTCTACGCGAACCTGAGCAGCTA	150 bp	58
		JUND-RT-AS	CTCGTCCTTGAGCGCAGCCAGGC		
PIK3CD	qRT-PCR	PIK3CD-RT-S	TCAACTCACAGATCAGCCTCC	73 bp	58
		PIK3CD-RT-AS	TTCACTTCTGGGTCGCACAAG		
CXCR4	qRT-PCR	CXCR4-RT-S	TGACGGACAAGTACAGGCTG	63 bp	58
		CXCR4-RT-AS	AGGGAAGCGTGATGACAAAGA		
P21	qRT-PCR	P21-RT-S	GCAGACCAGCATGACAGATTT	125 bp	58
		P21-RT-AS	GGATTAGGGCTTCCTCTTGGA		
P53	qRT-PCR	P53-RT-S	CCCAAGCAATGGATGATTTGA	91 bp	58
		P53-RT-AS	GGCATTCTGGGAGCTTCATCT		
GAPDH	qRT-PCR	GAPDH-RT-S	GAAGGTGAAGGTCGGAGT	226 bp	58
		GAPDH-RT-AS	GAGGATGGTGATGGGATTTC		
Alu	qRT-PCR	Alu-RT-S	GAGGCTGAGGCAGGAGAATCG	88 bp	60
		Alu-RT-AS	GTCGCCCAGGCTGGAGTG		
MALAT1-	PCR	512-S	AAACTCTGCAGTTTGGTC	500 bp	58
512		512-AS	AGCAGCACGGGCTGTCTG		
MALAT1-	PCR	4442-S	ATTGCAGATAAACTCATG	500 bp	58
4442		4442-AS	AACTTGCTTACACACAAC		
MALAT1-	PCR	5333-S	GCCAAAAAATTTTAAGCA	500 bp	58
5333		5333-AS	TCAATCCTGAAATCCCC		
MALAT1-	PCR	6649-S	CCATTCAGGATTTTGAATTG	474 bp	58
6649		6649-AS	CCCACCACCAGAAATGAAC		
MALAT1-	PCR	7038-S	GGAAATTTCTGCAGTTTTAA	500 bp	58
7038		7038-AS	TTCACCTGTTTTCCTCATTT		
MALAT1-	PCR	7575-S	TTATGGGACAATAGTATTG	500 bp	58
7575		7575-AS	GATGTGGCAGAGAAGTTG		

Supplemental data file-1. Top influenced-genes by *miR663a* in cDNA arrays

Supplemental data file-2. Sequences of *MALAT1* fragments inserted into pmiR-GLO vectors
